# Quercetin Nanocrystal Gel: A Novel Topical Therapeutic Strategy for Androgenetic Alopecia

**DOI:** 10.3390/pharmaceutics17091188

**Published:** 2025-09-12

**Authors:** Yaya Su, Yuwen Zhu, Lei Ren, Xiang Deng, Rui Song, Lingling Wu, Zhihui Yang, Hailong Yuan

**Affiliations:** Department of Pharmacy, Air Force Medical Center, PLA, Air Force Medical University, Beijing 100142, China; 173022331568@163.com (Y.S.);

**Keywords:** quercetin, glycyrrhizic acid, nanocrystals, skin penetration, androgenetic alopecia

## Abstract

**Purpose**: Androgenetic alopecia (AGA) is a common, chronic, non-cicatricial dermatological condition characterized by progressive miniaturization of hair follicles. Although AGA is a benign disorder, it has a considerable impact on patients’ quality of life and psychological health. The current treatment options often demonstrate limited efficacy and are frequently associated with undesirable side effects. This study aimed to co-mill two natural compounds, quercetin (QT) and glycyrrhizic acid (GL), to develop follicle-targeted nanocrystals (NCs), thereby enhancing local accumulation, improving the pathological follicular microenvironment associated with AGA, and promoting hair regrowth. **Methods**: QT nanocrystals (QT-NCs) were fabricated using a top–down wet media milling technique with GL as a bioactive stabilizer. The resulting QT-NCs were characterized regarding their particle size, crystallinity, morphology, and stability. The skin permeation properties of the QT-NCs were further evaluated in vitro, and their therapeutic efficacy was assessed in a dihydrotestosterone (DHT)-induced AGA mouse model. **Results**: The QT-NCs exhibited an irregular structure with a particle size ranging from 200 to 300 nm, demonstrating uniform dimensions and excellent storage stability. In vitro permeation studies revealed a 2.27-fold increase in cumulative penetration and a 2.47-fold enhancement in skin retention compared to raw QT. In the DHT-induced AGA mouse model, QT-NCs significantly reduced local DHT levels while concurrently modulating the follicular microenvironment, resulting in markedly improved therapeutic outcomes. Notably, when co-administered, QT and GL demonstrated synergistic pharmacological effects, suggesting potential combinatory benefits. **Conclusions**: This study presents the first demonstration of QT-NCs for AGA treatment, establishing a novel therapeutic strategy with substantial potential for clinical translation.

## 1. Introduction

Androgenic alopecia (AGA) is a highly prevalent form of nonscarring alopecia that affects both genders. The occurrence rate is as high as 80% in men and 50% in women [1]. Although AGA is not life-threatening, it imposes a significant psychological and emotional burden, substantially impacting patients’ quality of life [2]. The currently available pharmacological treatments, such as oral finasteride and topical minoxidil [3], exhibit limited efficacy and are often associated with undesirable side effects [4]. These limitations highlight the urgent need for safer and more effective therapeutic alternatives.

In recent years, increasing attention has been focused on plant-derived compounds as promising agents in the treatment of AGA, particularly flavonoids [5]. Quercetin (QT), a bioactive flavonoid abundantly present in various plant species [6], has demonstrated the ability to reactivate dormant hair follicles (HFs) upon topical application [7], stimulate keratinocyte proliferation, and promote perifollicular microvascular regeneration in mouse models [8]. However, the clinical utility of QT remains limited due to its poor aqueous solubility and low skin permeability, which result in suboptimal topical bioavailability [9]. Therefore, developing a delivery system that enhances QT’s dermal absorption and follicular targeting is essential.

Nanocrystals (NCs) represent an effective formulation strategy for enhancing the solubility and bioavailability of poorly water-soluble compounds. They typically range in size from 200 to 500 nm [10]. NCs offer several advantages, including high drug loading capacity, minimal excipient use, low toxicity, and broad formulation compatibility [11]. Additionally, NCs have demonstrated improved skin permeation and retention, enabling effective accumulation in hair follicles, making them highly suitable for topical drug delivery [12]. However, despite these advances, the use of nanocrystal technology for AGA treatment remains unexamined, presenting a major opportunity for innovative therapy development. Despite these advantages, the application of nanocrystal technology in the context of AGA remains underexplored.

Stabilizers play a crucial role in the preparation of NCs by preventing particle aggregation and ensuring colloidal stability [13]. Compared to traditional surfactants, plant-derived bioactive compounds have gained increasing attention due to their dual role as both stabilizers and therapeutic agents [14]. Glycyrrhizic acid (GL), a natural triterpenoid saponin extracted from licorice roots, has been extensively studied for its ability to improve the solubility and bioavailability of hydrophobic compounds. In addition to its stabilizing properties, GL also exhibits hair-growth-promoting effects, potentially through upregulation of α-melanocyte-stimulating hormone (α-MSH) and interleukin-10 (IL-10) [15,16].

**Scheme 1:**
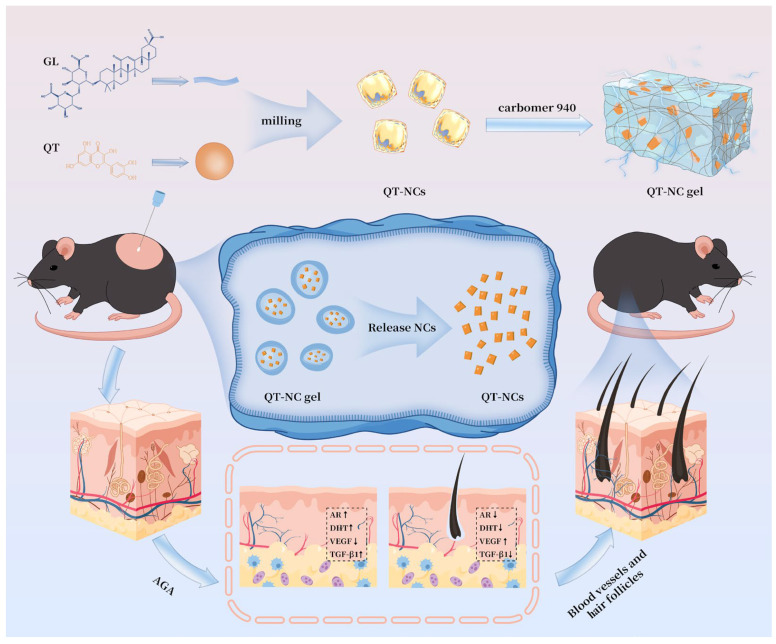
QT-NC gel: a novel therapeutic strategy for AGA.

Inspired by the above findings, QT-NCs were fabricated through a top–down approach with GL incorporation to enhance the topical bioavailability of QT while achieving the co-delivery of both QT and GL. Concurrently, the therapeutic efficacy of QT-NCs against AGA was systematically evaluated, and the pharmacological mechanisms of individual components were further elucidated. This investigation provides both a feasible research strategy and a potential therapeutic approach to address the current clinical challenges in AGA management. The experimental design is illustrated in Scheme 1.

## 2. Materials and Methods

### 2.1. Materials

Quercetin (QT, purity > 98%) was purchased from Shaanxi Zelang Biotechnology Co., Ltd. (Shaanxi, China). Carbopol^®^940 was obtained from Guangzhou Baiyu Biotechnology Co., Ltd. (Guangzhou, China). Triethanolamine was purchased from Tianjin Damao Chemical Reagent Factory (Tianjin, China). Tween 80 was sourced from Fuchen Chemical Reagent Co., Ltd. (Tianjin, China). Dihydrotestosterone (DHT, purity > 98%) was purchased from Tianjin Nuoer Pharmaceutical Technology Co., Ltd. (Tianjin, China). Minoxidil tincture (2%) was obtained from Shanxi Zhendong Ante Biopharmaceutical Co., Ltd. (Beijing, China). Glycyrrhizic acid (purity > 95%) was provided by Beijing Century Aoke Biotechnology Co., Ltd. (Beijing, China). DHT, 5α-reductase (5AR), transforming growth factor β1 (TGF-β1), and vascular endothelial growth factor (VEGF) kits were purchased from Shanghai Enzyme-linked Biotechnology Co., Ltd. (Shanghai, China). The AR, CD31, and Ki67 antibodies were purchased from Wuhan Sevier Biotechnology Co., Ltd. (Wuhan, China).

### 2.2. High-Performance Liquid Chromatography (HPLC) Analysis

HPLC analysis of QT was conducted using an LC-20 CE system (Shimadzu Corp, Kyoto, Japan) with an InterSustain C18 column (250 × 4.6 mm, 5 µm). The analyses were performed with a mobile phase consisting of methanol/0.1% phosphoric acid (59:41, *v*/*v*) at a flow rate of 1.0 mL/min under the following conditions: column temperature of 30 °C, detection wavelength of 375 nm, and an injection volume of 20 µL. Samples were filtered through a 0.22 µm filter before analysis. The quantitative determination method for QT was developed by modifying established protocols from previous studies [17]. This optimized method demonstrated excellent linearity (R^2^ = 0.9999, with a regression equation of A = 93,463C + 11,078) over a concentration range of 0.2–80.0 μg/mL, along with high specificity. Additionally, it exhibited satisfactory precision and repeatability (Figures S1 and S2, Tables S1 and S2).

### 2.3. Preparation of QT-NCs

QT-NCs were produced through wet media milling [18]. Initially, QT (0.1 g) was dispersed in 20 mL of deionized water under continuous stirring. Subsequently, varying mass ratios of GL were added and thoroughly dissolved under agitation. An equal volume of zirconium oxide beads (diameter 0.4–0.6 mm) was then introduced, and the mixture was subjected to wet milling using a DF101D magnetic stirrer (Gongyi Yuhua Instrument Co., Ltd., Henan, China) at 25 °C. The process parameters, including the mass ratio of QT to GL (8:1, 4:1, 2:1, 1:1, and 1:2, *w*/*w*), milling speed (200, 400, 600, 800, and 1000 rpm), and milling time (1.0, 2.0, 3.0, 4.0, and 5.0 h), were systematically optimized, as summarized in Table 1. The results were evaluated based on the particle size (PS), polydispersity index (PDI), and stability index (SI).

### 2.4. Preparation of QT-NC Gel

Carbomer 940 (0.5 g) was accurately weighed and dissolved into 50 mL of ultrapure water, with continuous stirring until a uniform dispersion formed. Then, the pH was adjusted to neutral using triethanolamine. After that, the gel was used by mixing with NC suspensions at a ratio of 1:1 (*v*/*v*), and the pH was adjusted to approximately 6.5 to create QT-NC gel, which was stored at 25 °C in a dark, stable environment for later use [19].

### 2.5. Physiochemical Characterization of QT-NCs

#### 2.5.1. PS, PDI, and ζ-Potential of QT-NCs

QT-NCs were diluted with distilled water, and then, the PS and PDI were measured using a nanoparticle size analyzer (Jinan Winner Particle Instrument Stock Co., Ltd., Shandong, China). ζ-potential was determined using a JS94H zeta potential analyzer (Shanghai Zhongchen Digital Technic Apparatus Co., Ltd., Shanghai, China).

#### 2.5.2. Stability

The stability of QT-NCs was assessed using the SI, calculated with modifications to a previously published method [20] as follows:

SI (%) = PS_c_/PS_0_ × 100% (1)

where PS_0_ represents the mean particle size of QT-NCs before centrifugation, and PS_c_ denotes the particle size of the supernatant after centrifugation (3000 rpm, 15 min); the closer the SI is to 100%, the better the stability of the NCs.

Additionally, QT-NCs were stored in vials tightly sealed with polystyrene caps and underwent storage stability testing at 4 °C and 25 °C. The particle size of the stored samples was assessed over a 7-day period [21].

#### 2.5.3. Microstructural Characterization

Samples were precooled at −80 °C for 2.0 h before lyophilization (FD1A-50, Biocool, Beijing, China) for 24 h. The freeze-dried samples were sputter-coated with gold prior to scanning, and the structures of both QT and QT-NCs were observed using scanning electron microscopy (SEM) (Hitachi S-4800, Tokyo, Japan) at an acceleration voltage of 5 kV [22]. Morphological characterization of QT-NCs was conducted using transmission electron microscopy (TEM) (JEM-2100F, JEOL, Tokyo, Japan). Samples were diluted with distilled water and deposited onto copper grids before air-drying. TEM imaging was performed at an accelerating voltage of 80 kV [23].

#### 2.5.4. X-Ray Diffractometry (XRD)

XRD analysis was conducted to characterize the crystalline structure of lyophilized QT, GL, and QT-NCs using a Rigaku SmartLab SE diffractometer (Rigaku Corporation, Tokyo, Japan). Scans were performed with a Cu source of radiation at 40 kV voltage and with 25 mA current over the 2θ range of 3–60° at a scanning rate of 4°/min [24].

#### 2.5.5. Differential Scanning Calorimetry (DSC)

Thermal properties were analyzed using DSC (200 F3, Netzsch, Selb, Germany). Samples (5 mg) of lyophilized QT, GL, and QT-NCs were sealed in aluminum crucibles and heated from 50 °C to 350 °C at 10 °C/min under a nitrogen atmosphere [25].

#### 2.5.6. Fourier Transform Infrared (FTIR) Spectroscopy 

The lyophilized samples were thoroughly mixed with KBr powder and subsequently compressed into tablets. The FTIR spectra of QT, GL, and QT-NCs were captured with a Nicolet 6700 spectrometer (Thermo Fisher Scientific, Waltham, MA, USA) across the range of 4000–500 cm^−1^ at a resolution of 4 cm^−1^ [21].

### 2.6. Rheological Analysis

The static rheological properties and viscoelastic properties of the gel were measured at 25 °C using an AR 2000 ex rotational rheometer (TA Instruments, New Castle, DE, USA) with 40 mm parallel stainless steel plates (1 mm gap). The apparent viscosity was determined over shear rates of 0.1–100 s^−1^, and data analysis was conducted using the power-law model. Frequency sweep tests were conducted at 0.5% strain across an angular frequency range of 0.63–79.1 rad/s to determine the storage (G′) and loss (G″) moduli [26].

### 2.7. Ex Vivo Skin Permeation and Deposition Analysis

Abdominal skin was harvested from 6-week-old male Sprague Dawley rats, stored at −20 °C. In vitro transdermal experiments were conducted using an automated transdermal diffusion system (LOGAN, SYSTEM 918-12, Somerset, NJ, USA). Pre-treated rat abdominal skin was soaked in PBS for 30 min, dried with filter paper, and prepared for use. The receptor fluid was a PBS/ethanol solution (4:1, *v*/*v*) with 2.0% Tween 80. The water bath temperature was maintained at 37.0 ± 0.5 °C, and the stirring speed was set to 800 rpm. Samples of 1 mL were collected from the receptor compartment at 6, 12, 18, 24, 30, 36, 42, and 48 h, with an equal volume of pre-warmed blank receptor fluid replenished after each sampling. The samples were filtered through a 0.45 μm membrane and analyzed by HPLC to determine the drug content.

At the end of the experiment (48.0 h), the skin was collected and washed three times with distilled water. The skin samples were cut into small pieces and extracted with 2.0 mL of methanol using ultrasonication (60 min, 500 W). The extracts were then filtered through a 0.22 μm membrane, and the QT concentration in the skin was determined [27].

### 2.8. In Vivo Study

#### 2.8.1. AGA Model Establishment and Treatment

All animal procedures were performed in compliance with protocols approved by the Ethics Committee of the Air Force Medical Center, PLA. Healthy male C57BL/6 mice (7-week-old, SPF-grade, Home-SPF (Beijing, China) Biotechnology Co., Ltd. (Ethics No. 2025-15-PJ01)) were acclimatized for one week before experimentation. The laboratory environment was maintained under the following controlled conditions: ambient temperature of 20–26 °C with daily fluctuations not exceeding 4 °C, relative humidity between 40% and 70%, air exchange rate of 15–20 times per hour, airflow velocity below 0.2 m/s, and pressure gradients ranging from 20 to 50 Pa. The dorsal hair of selected mice was uniformly removed using electric clippers, followed by depilatory cream application. Mice were randomly allocated into six groups based on body weight (*n* = 6): blank control, model group, high-/medium-/low-dose QT-NC groups (QT-NCs-gel-H, QT-NCs-gel-M, and QT-NCs-gel-L), and minoxidil group. An androgenetic alopecia model was established through daily topical administration of 0.2% DHT (*w*/*v*) solution [28]. The 0.2% DHT solution was applied to all groups except the controls, with respective therapeutic agents administered topically 1.0 h post application for 20 consecutive days. Dorsal skin alterations were photographically monitored throughout the intervention period. The experiment was concluded upon achieving hair coverage exceeding 80% in the normal control group, at which point euthanasia was performed and skin tissues were collected for subsequent analysis.

#### 2.8.2. Evaluation of in Vivo Hair Growth

The changes in skin color during the treatment were observed and photographed. The time of skin pigmentation on the back of the mice was recorded. ImageJ Version 1.54p (NIH, Bethesda, MD, USA) software was used to measure the hair-covered area on the back skin on days 8, 12, 16, and 20, and the percentage of hair coverage was calculated as follows:

Hair coverage (%) = Area (hair recovery)/Area (total) × 100%(2)

where Area (hair recovery) represents the hair recovery area of the dorsal shaved skin after treatment, and Area (total) is the entire shaved dorsal area.

SEM was used to analyze the surface morphology of newly regrown hair collected from different skin regions across all experimental groups. Representative hair samples were mounted on stubs, sputter-coated with gold, and imaged at suitable magnifications. The diameter of the hair shaft was then measured quantitatively using ImageJ software (NIH, USA), calibrated with a standardized scale bar [29].

#### 2.8.3. Histology and Immunofluorescence

A part of the skin tissue collected 20 days after hair removal was fixed in tissue fixative solution for 24 h and processed into paraffin sections for hematoxylin and eosin (H&E), Ki67, CD31, and AR staining analyses. Images of the sections were captured using a digital slide scanner (Kf-pro-020, KFBIO, Wuhan, China). The skin thickness of mice in each group was measured with ImageJ software based on the sections.

#### 2.8.4. Determination of DHT, 5-AR, VEGF, and TGF-β1 in Skin

ELISA quantification of DHT, 5AR, VEGF [30], and TGF-β1 levels in skin tissue was performed following standard procedures. Dorsal skin samples were washed with ice-cold PBS, mechanically homogenized after mincing, and centrifuged at 5000 rpm for 10 min. The resulting supernatant was analyzed according to the manufacturer’s ELISA protocol.

### 2.9. Data Analysis

All experimental data are presented as the mean ± standard deviation (SD). Statistical analyses were conducted using GraphPad Prism 8.0.2 and SPSS 21.0 (IBM, USA). Intergroup differences were assessed by one-way ANOVA followed by post hoc tests or Student’s *t*-test, as appropriate. A *p* value of less than 0.05 was considered statistically significant.

## 3. Results

### 3.1. Preparation of QT-NCs

**Figure 1:**
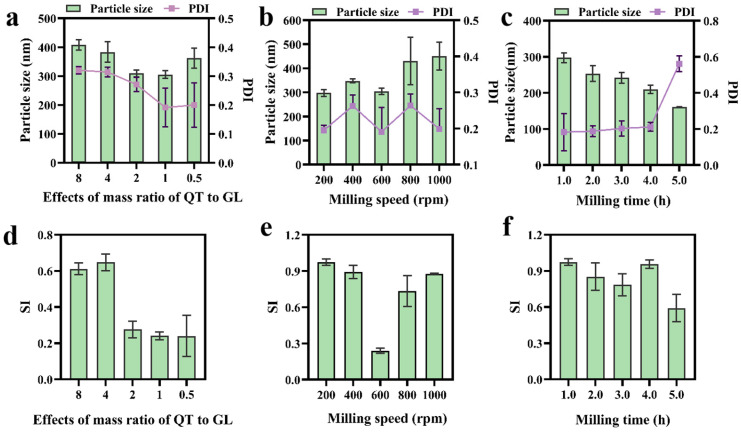
The effects of (**a**,**d**) the mass ratio of QT to GL, (**b**,**e**) milling speed, and (**c**,**f**) milling time on the particle size, PDI, and SI of QT-NCs, *n* = 3.

**Figure 2:**
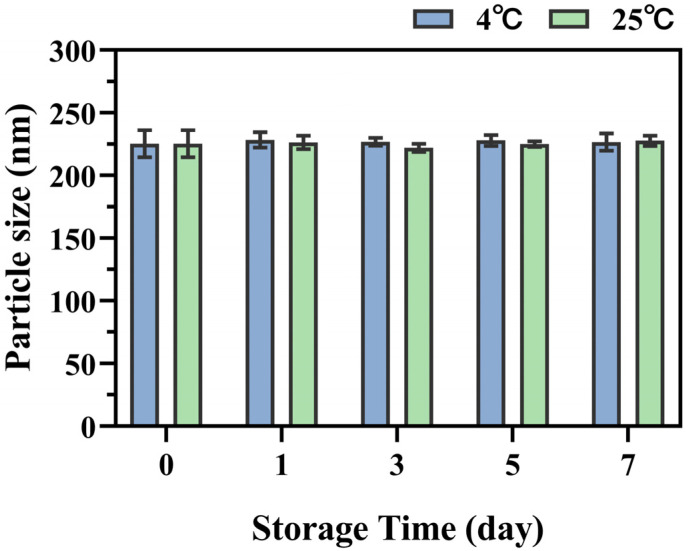
Stability testing of QT-NCs over 7 days.

QT-NCs were prepared using a wet media milling technique, an eco-friendly approach that eliminates the need for organic solvents [31]. To achieve the desired PS distribution, the mass ratio of QT to GL, milling speed, and milling time were systematically optimized. As shown in Figure 1a,d, the PS, PDI, and SI were significantly influenced by the QT∶GL mass ratio. As the QT∶GL mass ratio increased, both the particle size and PDI initially decreased and then increased, with the smallest particle size and PDI observed at a 1:1 mass ratio. Therefore, this ratio was selected for further optimization. The influence of the milling speed on the PS, PDI, and SI is shown in Figure 1b,e. While the milling speed had minimal impact on the PDI, it significantly affected the PS and SI. At 200 rpm, QT-NCs exhibited the smallest PS and the highest SI; thus, 200 rpm was chosen as the optimal milling speed. Figure 1c,f illustrate the effects of the milling time. The milling duration affected all parameters to varying degrees. A milling time of 4 h resulted in the smallest PS and the highest SI. Accordingly, the optimized preparation conditions were determined to be a 1:1 mass ratio of QT to GL, a milling speed of 200 rpm, and a milling time of 4 h. Under these conditions, the resulting QT-NCs exhibited a particle size of 208.70 ± 6.94 nm, a PDI of 0.179 ± 0.043, an SI of 0.955 ± 0.035, and a ζ-potential of −33.41 ± 0.14 mV. Furthermore, the QT-NCs exhibited outstanding storage stability, showing no significant change in particle size following 7-day storage at both 4 °C and 25 °C (Figure 2).

### 3.2. SEM and TEM

**Figure 3:**
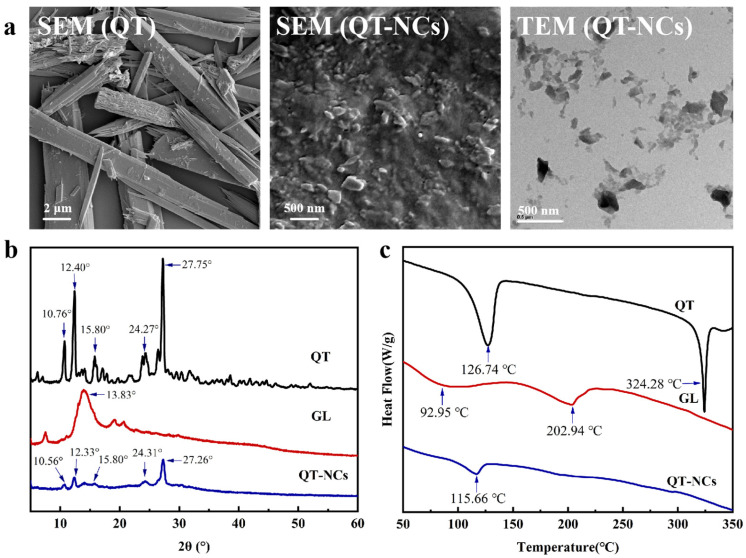
(**a**) SEM images at 5000× magnification of QT and 2000× magnification of QT-NCs and TEM photograph of QT-NCs. (**b**) XRD pattern and (**c**) DSC curve of QT, GL, and QT-NCs.

Microstructural characterization revealed significant morphological changes following the preparation of nanocrystals. Unprocessed QT exhibited distinct angular and rod-shaped crystals (10–40 μm) with a heterogeneous distribution (Figure 3a). Post-milling analyses using SEM and TEM confirmed successful particle size reduction to 200–300 nm, consistent with the particle size analysis. The original crystalline morphology transitioned into irregular fragments and short rods, a result of mechanical fragmentation by zirconia beads during the wet milling process [32].

### 3.3. Physiochemical Characterization of QT-NCs

The changes in the crystalline structure of QT before and after milling into QT-NCs were analyzed using XRD (Figure 3b). QT exhibited sharp diffraction peaks at 10.76°, 12.40°, 15.81°, 24.27°, and 27.25°, indicating high crystallinity, which is generally consistent with previous reports. A weak diffraction peak of GL was also observed at 13.83° [33]. However, after milling, the diffraction peaks of QT were significantly weakened, and the characteristic peak of GL disappeared, indicating a reduction in crystallinity. This phenomenon could be attributed to the PS reduction in QT to the nanoscale during wet milling or to the disruption of the original crystal packing structure, possibly due to hydrogen bonding or hydrophobic interactions between QT and GL, resulting in alterations to the diffraction pattern.

The DSC results are presented in Figure 3c. QT exhibited two distinct endothermic peaks at 126.74° [33] and 324.28° [34], which correspond to the melting of its crystalline form. After nanocrystal formation, the intensity of these melting peaks was significantly diminished or even completely absent, suggesting a transition of QT from a crystalline to an amorphous state [33]. Furthermore, the characteristic endothermic peak of GL at 202.94 °C disappeared, most likely due to the milling process and potential intermolecular interactions between QT and GL [35,36].

**Figure 4:**
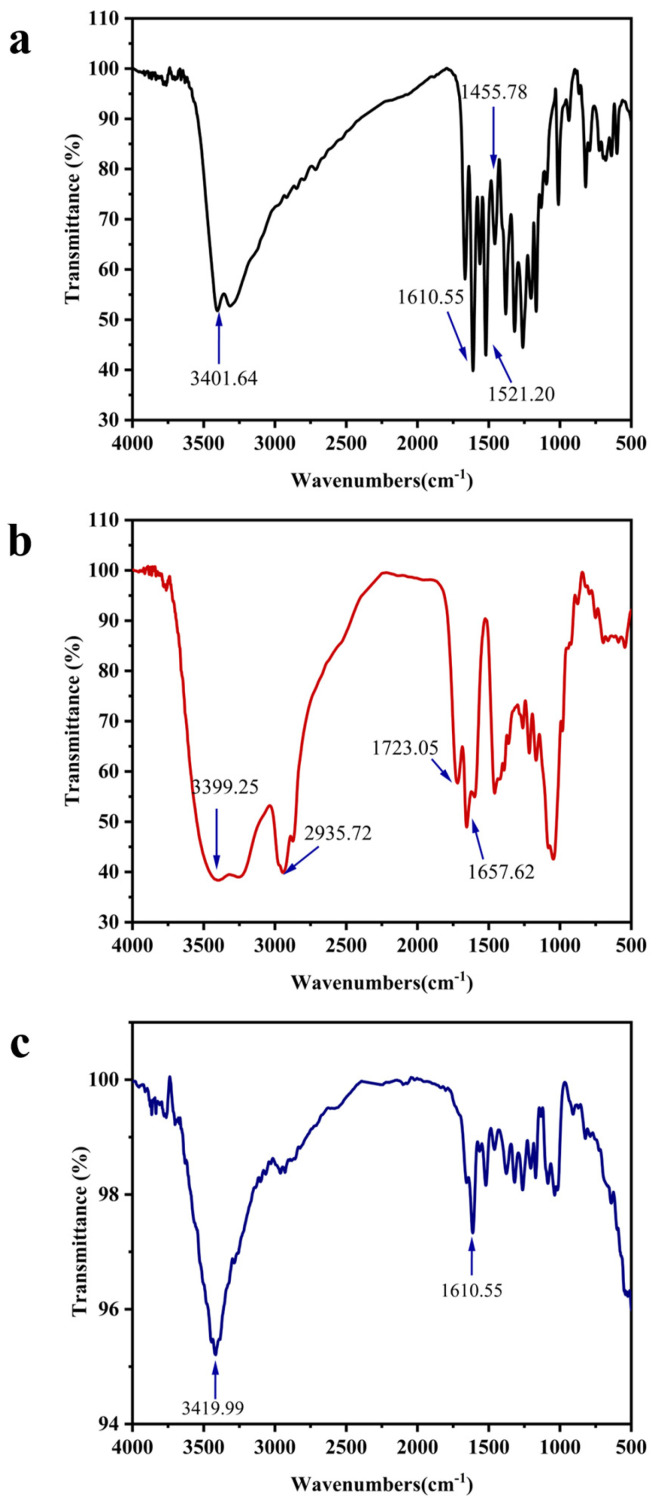
FTIR spectra of (**a**) QT, (**b**) GL, and (**c**) QT-NCs.

To further investigate the interaction mechanism between QT and GL, FTIR spectroscopy analysis was performed (Figure 4). The infrared spectrum of QT (Figure 4a) displayed characteristic absorption peaks at 1455.78 and 1521.20 cm^−1^, corresponding to the stretching and bending vibrations of the phenyl rings. A peak at 3401.64 cm^−1^ was assigned to O–H stretching vibrations, while a peak at 1610.55 cm^−1^ was attributed to C=O stretching vibrations [33]. For GL (Figure 4b), an O–H stretching peak was observed at 3419.99 cm^−1^ [37], and a C=O stretching vibration of the carboxyl group was detected at 1657.62 cm^−1^, consistent with its known chemical structure [16]. The characteristic peaks of both QT and GL were retained in the QT-NCs (Figure 4c), although their intensities were reduced. Notably, the O–H stretching peak of QT at 3401.64 cm^−1^ exhibited a blue shift, providing direct evidence of hydrogen bond formation between QT and GL molecules [38].

### 3.4. Rheological Analysis

**Figure 5:**
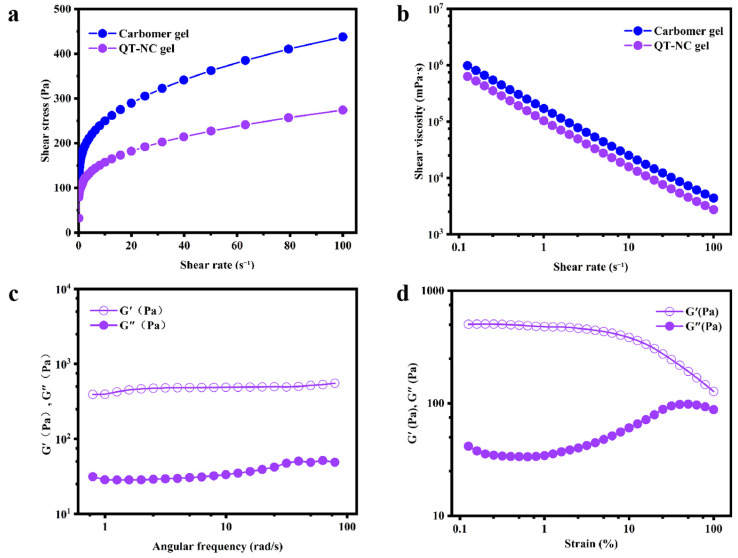
The rheological characteristics. (**a**) The relationship between the shear stress and shear rate. (**b**) The relationship between the shear viscosity and shear rate. (**c**,**d**) The dynamic rheological properties of gel.

Figure 5a illustrates the relationship between the shear stress and shear rate, demonstrating that the gel samples exhibit non-Newtonian flow behavior. The shear-thinning segment of the flow curve aligns well with the power-law model, showing a strong correlation, as each fitting equation has an R^2^ greater than 0.97. Table 2 summarizes the rheological parameters K and n, which represent the apparent viscosity and shear-thinning characteristics of the gel. Figure 5b shows that as the shear rate increases, the viscosity of the gel decreases rapidly, further confirming its shear-thinning behavior [39]. These results suggest that the carbomer-based gel exhibits pseudoplastic behavior, with flow properties strongly influenced by the polymer’s molecular structure. In the pseudoplastic region, as the shear rate increases, the entangled coils deform and stretch, occupying less space and thereby reducing the gel’s flow resistance [26]. Consequently, as the shear rate increases, the viscosity of the gel decreases.

The dynamic rheological properties of the QT-NC gel are shown in Figure 5c,d. Throughout the measured frequency range, the G′ of the QT-NC gel remains consistently higher than G″, correlating with the increasing scanning frequency. The gel’s elasticity was evaluated using the frequency sweep method. In Figure 5d, rheological testing demonstrated the mechanical properties of the QT-NC gel, exhibiting gel-like and elastic behavior (G′ > G″) [40]. Notably, as the scanning frequency increases, there is little change in G′ and G″, indicating that the sample maintains its elasticity and stability [19]. These findings suggest that the gel can effectively diffuse and maintain its structure without phase separation, making it suitable for skin applications.

### 3.5. In Vitro Percutaneous Study

**Figure 6:**
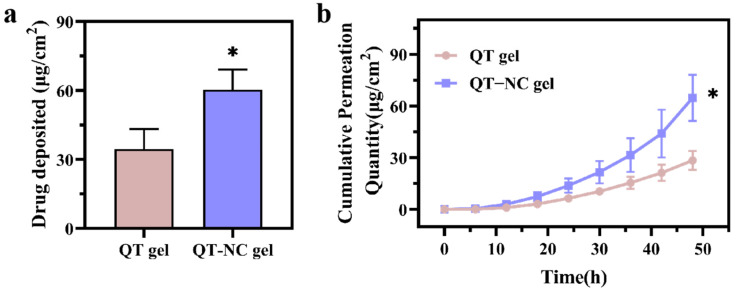
Ex vivo permeation (**a**) and QT deposition (**b**) of QT gel and QT-NC gel. *n* = 3; * *p* < 0.05.

The cumulative amount of QT per unit area (μg/cm^2^) was plotted against time (h) to show the trend of QT accumulation across different samples (Figure 6b). At 48 h, the cumulative amount of QT per unit area for QT gel was 28.48 μg/cm^2^. At the same time, for QT-NC gel, it was 64.79 μg/cm^2^, which is 2.27 times higher than that of QT gel. This suggests that the formation of NCs can improve the overall penetration of QT. After 48 h of in vitro transdermal penetration, the residual QT accumulated in the skin was measured, as shown in Figure 4a. The retention of QT gel was 8.88 μg/cm^2^, while that of QT-NC gel was 22.01 μg/cm^2^, which is 2.47 times higher than that of QT gel. In the QT deposition study, QT-NC gel demonstrated high penetration and skin retention. Additionally, the relevant permeation parameters (Table 3) demonstrated that both the steady-state flux (J_ss_) and permeation coefficient (K_p_) of QT-NC gel were significantly increased compared to QT gel, with a permeation enhancer index (EI) of 2.14, indicating a 1.14-fold improvement in permeation efficiency. These results demonstrate that QT-NC formation significantly enhances the transdermal delivery of QT, establishing an effective topical administration strategy, which corroborates existing findings in the field [13].

### 3.6. QT-NC Gel Encourages Hair Growth

**Figure 7:**
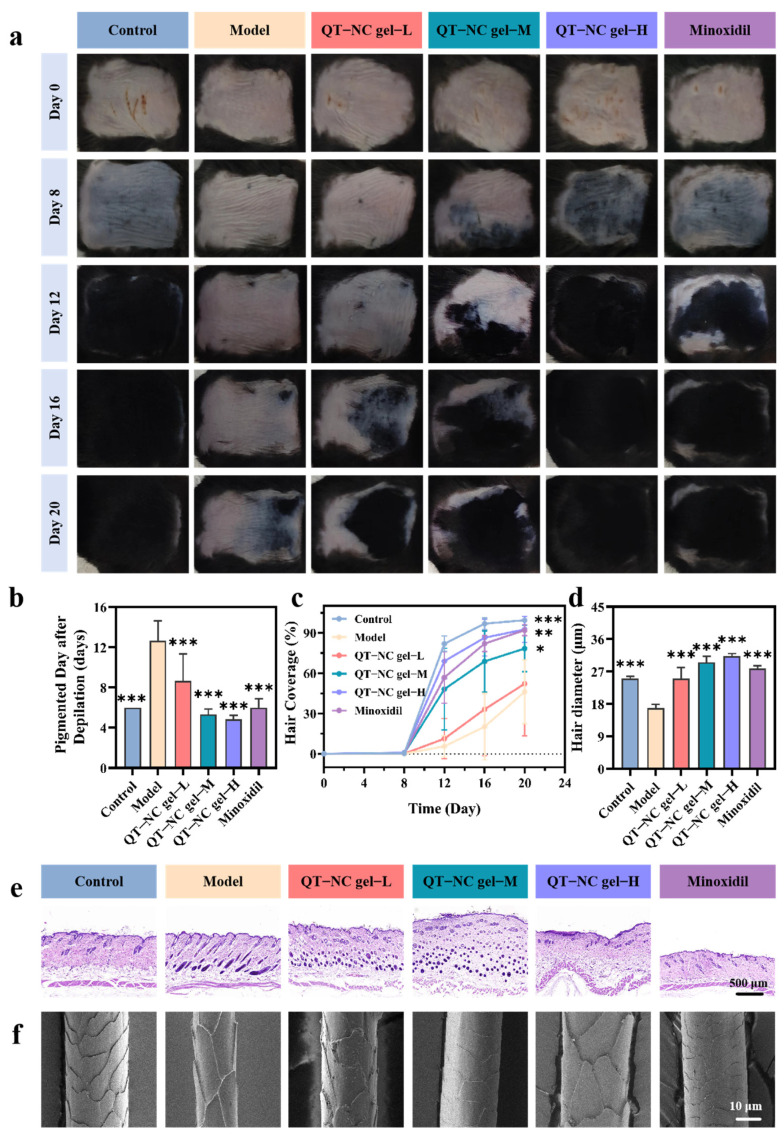
(**a**) Representative images of skin changes over 20 days. (**b**) Melanin deposition over time (*n* = 6). (**c**) Hair coverage on days 0, 8, 12, 16, and 20 (*n* = 6). (**d**) Hair diameter (*n* = 3). (**e**) Representative H&E-stained images. (**f**) Representative SEM images of hair. * *p* < 0.05; ** *p* < 0.01; *** *p* < 0.001.

In the in vivo study, differences in skin color and hair growth among the treatment groups were evaluated through visual inspection over a 20-day treatment period (Figure 7a). The skin color of mice typically reflects the hair growth cycle, with pink indicating the telogen phase of hair follicles and gray-black indicating the anagen phase. Therefore, the time it takes for the skin to darken after depilation can serve as an indicator of the transition from the telogen to the anagen phase [30]. Without any treatment, the dorsal skin of the mice was mostly pink and smooth, indicating that the hair follicles were in the resting phase [30,41]. As shown in Figure 7b, the time to pigmentation was significantly shorter in all treatment groups compared to the model group. By day 8, the control, minoxidil, and medium-dose groups began to show pigmentation, and the high-dose group started to develop a small amount of new hair. After 12 days of treatment, the dorsal skin color of all groups, except the model group, had changed from pink to gray-black, accompanied by varying degrees of hair regrowth. By day 20, the depilated areas in both the high-dose and minoxidil groups were almost completely covered with hair, while the medium-dose group exhibited nearly 80% hair coverage, which was significantly higher than that of the model group (Figure 7c).

The histopathological examination of skin tissues using H&E staining is shown in Figure 7e. Both the medium- and low-dose QT-NC gel groups exhibited HFs in the anagen phase. Notably, spontaneous entry into the telogen phase was observed in the blank control, high-dose QT-NC gel, and minoxidil groups, indicating restoration of normal hair cycle progression in these treatment groups. This phenomenon was consistent with previous research findings [42].

SEM images of newly grown hair (Figure 7f) revealed that the regenerated hair in the model group had a smaller diameter and fewer cuticles. In contrast, the treatment groups exhibited varying degrees of increased hair diameter and more complete cuticle formation. The results indicated that by day 20, all treatment groups had a significantly larger hair diameter compared to the model group (Figure 7d), with the high-dose group’s regenerated hair diameter slightly surpassing that of the minoxidil group. These findings suggest that the treatment effectively shortens the hair growth cycle and accelerates the transition of HFs into the anagen phase.

**Figure 8:**
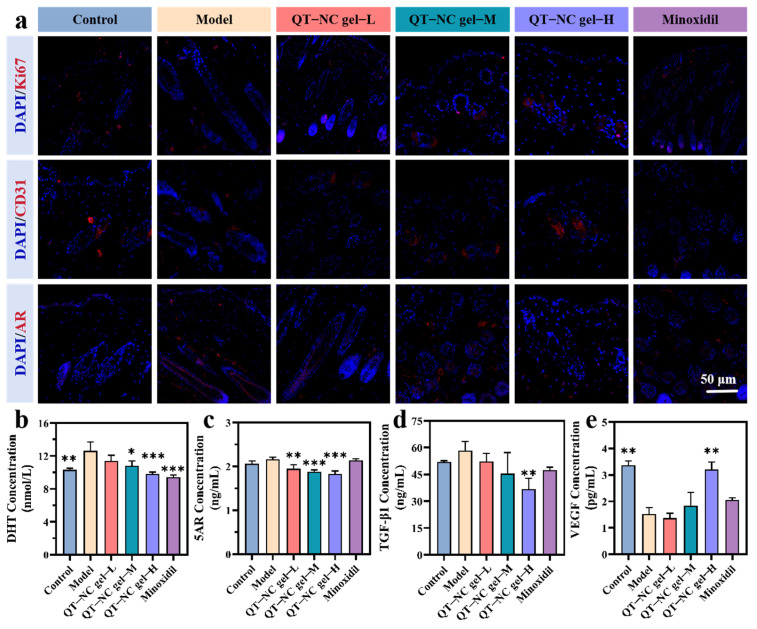
(**a**) Representative images of Ki67, CD31, and AR immunofluorescence staining in skin tissue from different groups on day 20 post epilation. (**b**) The concentration of DHT, (**c**) 5AR, (**d**) TGF-β1, and (**e**) VEGF in skin by ELISA. *n* = 3; * *p* < 0.05; ** *p* < 0.01; *** *p* < 0.001.

The transition of HFs from the telogen phase to the anagen phase involves rapid cell proliferation. Ki67, a marker of cell proliferation in dorsal skin, is primarily expressed in the nuclei of cells [43]. As shown in Figure 8a, a high level of Ki67 expression was observed in the HFs of the depilated area in the treatment group on day 20. Additionally, CD31, a specific marker for vascular endothelial cells, indicates the distribution of blood vessels within the tissue. CD31 immunofluorescence staining on day 20 revealed almost no positive CD31 expression around the HFs in the model group, whereas the treatment group exhibited varying degrees of angiogenesis.

The study identified elevated DHT and 5AR levels and AR overexpression as two primary pathogenic factors in AGA. Consequently, cutaneous DHT and 5AR concentrations were quantified alongside follicular AR expression analysis (Figure 8b,c). A dose-dependent reduction in both DHT and 5AR content was observed, whereas minoxidil only demonstrated significant DHT suppression. These findings showed complete concordance with the immunofluorescence results presented in Figure 8a. Furthermore, TGF-β1 (Figure 8d), a negative regulator of hair growth, was also reduced in the skin tissue of the treatment groups to varying extents, with the high-dose group showing a significant decrease compared to the model group. VEGF shows strong angiogenic activity and affects the development of microvessels in HFs [44]. As shown in Figure 8e, VEGF expression in the skin treated with the high-dose gel was significantly increased compared to the model group, consistent with the CD31 immunofluorescence staining results, further confirming that the high-dose QT-NC gel has pro-angiogenic activity.

### 3.7. QT and GL Promoted Hair Growth

**Figure 9:**
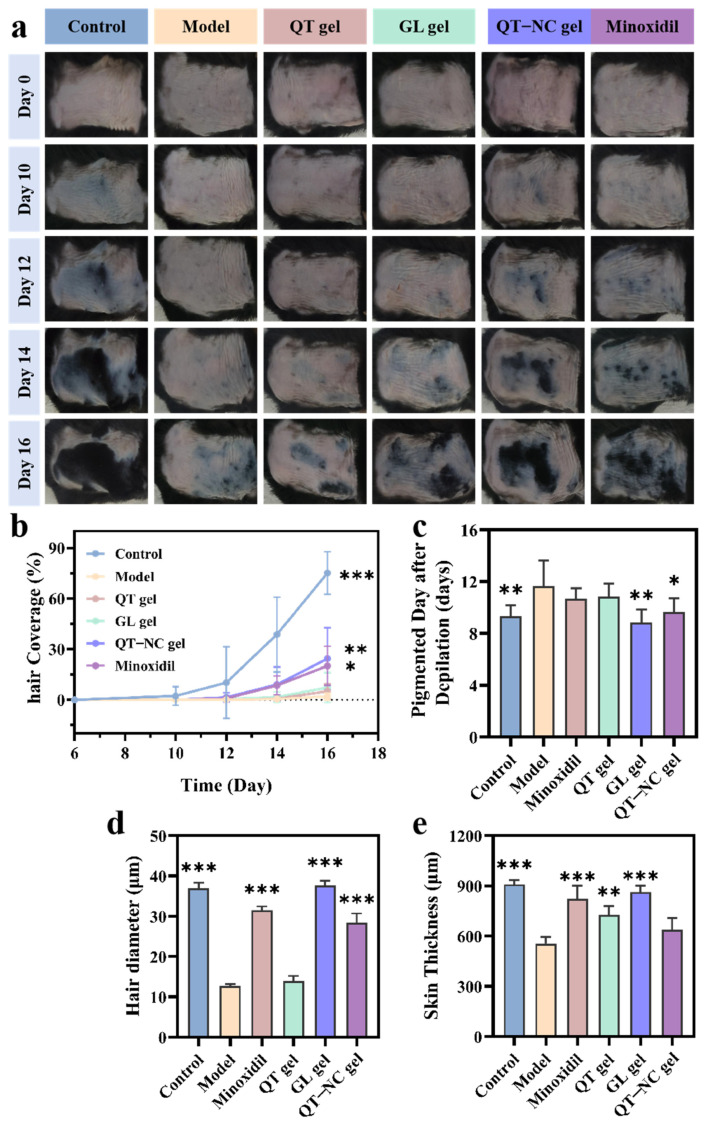
(**a**) Representative images of skin changes over 16 days (*n* = 6). (**b**) Hair coverage on days 0, 10, 12, 14, and 16 (*n* = 6). (**c**) Melanin deposition over time (*n* = 6). (**d**) Hair diameter (*n* = 6). (**e**) Skin thickness measurement (*n* = 3). * *p* < 0.05; ** *p* < 0.01; *** *p* < 0.001.

In the 16-day in vivo study, differences in skin color and hair growth among the treatment groups were assessed through visual inspection. On day 1, most of the dorsal skin of the mice appeared pink and smooth, indicating that the hair follicles were in the telogen phase. By day 16, varying degrees of regenerated hair coverage were observed in the shaved areas, except for in the model group (Figure 9a). Specifically, after 12 days of treatment, the dorsal skin color of the mice shifted from pink to gray in all groups except the model group. However, the changes in the QT gel and GL gel groups were less prominent compared to those in the QT-NC gel and minoxidil groups. Figure 9c shows different levels of pigmentation in the QT-NC gel and minoxidil groups on days 10 and 9, respectively, signaling the onset of the anagen phase. By day 12, initial regrowth of black hair was visible on the gray skin. On day 14, hair began to cover parts of the depilated areas in all groups except the untreated group. By day 16, the hair coverage results (Figure 9b) showed significant differences between the QT-NC gel and minoxidil groups compared to the model group. These results indicate that both QT gel and GL gel can promote the transition of skin from the telogen phase to the anagen phase. When used together, their promoting effects are comparable to those of minoxidil.

**Figure 10:**
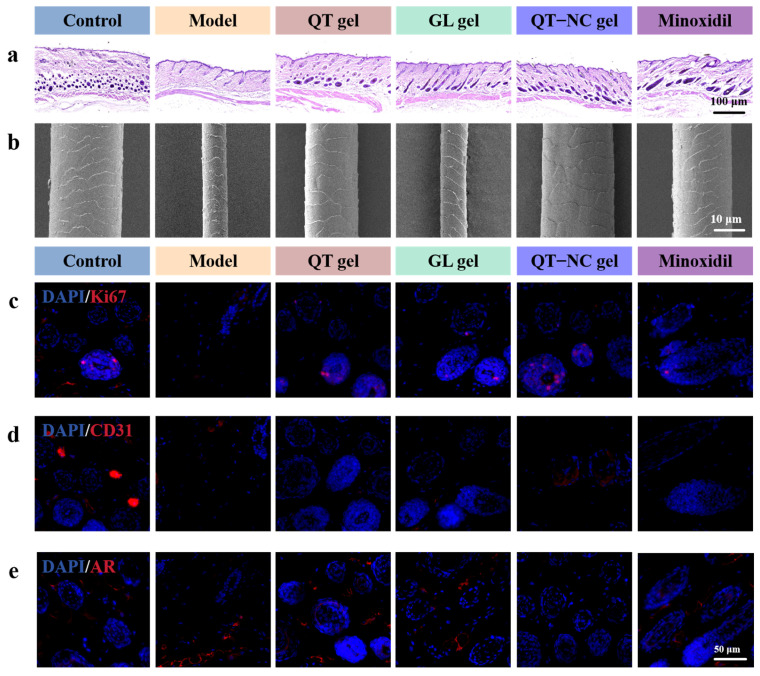
(**a**) Representative H&E-stained images. (**b**) Representative SEM images of hair. (**c**) Representative images of Ki67, (**d**) CD31, and (**e**) AR immunofluorescence staining of skin tissue from different groups on day 16 post epilation.

The dorsal skin sections were examined using H&E staining (Figure 10a). Longitudinal sections of the pink skin from the model group showed miniaturization and atrophy of HFs, along with a decrease in overall skin thickness (Figure 9e), indicating that the HFs remained in the telogen phase. In comparison to the model group, the skin treated with various formulations exhibited a greater number of HFs, with most of them in the anagen phase, and increased skin thickness (Figure 9e). The gel group showed significant improvement over the model group. In terms of skin thickness restoration, the gel group demonstrated a notable advantage over the minoxidil group. Furthermore, after treatment with QT-NC gel, the hair bulb portion of the dorsal skin HFs increased significantly, the hair shafts became thicker, and more melanin accumulated compared to in the QT gel and GL gel groups, approaching levels observed with minoxidil.

SEM images of newly grown hair (Figure 10b) revealed that the regenerated hair diameter in the model group was smaller, whereas the diameter in the treatment groups increased to varying extents. The results indicated that the hair diameter in the QT-NC gel group on day 16 was significantly larger than that in the model group (Figure 9d) and comparable to that in the minoxidil group, with no significant difference in the hair cuticle length. These findings further demonstrate that both QT gel and GL gel can promote the transition of skin from the telogen phase to the anagen phase. When used together, their effects are comparable to those of minoxidil and, in some aspects, even slightly superior.

As shown in Figure 8c, a high level of Ki67 expression was clearly observed in the HFs of the depilated area in the treatment group on day 16. CD31 immunofluorescence staining of the skin tissue on day 16 revealed almost no positive CD31 expression around the HFs in the model group, while the treatment group exhibited varying degrees of angiogenesis (Figure 10d). The AR expression level in HFs (Figure 10e) showed that the model group had high AR expression, whereas AR expression was nearly absent in the treatment groups.

## 4. Discussion

AGA remains a prevalent and psychologically distressing dermatological condition with limited effective treatment options [45]. Current first-line agents, such as minoxidil and finasteride, often exhibit suboptimal efficacy, limited target specificity, and adverse effects that hinder long-term patient adherence [8,46]. In this context, phytochemical-based therapeutics have garnered increasing attention as safer alternatives with multi-target regulatory potential [47].

This study successfully developed a QT-NC gel to address the limitations of poor solubility and bioavailability inherent in QT. Using a top–down wet milling method, QT was reduced to nanoscale particles with irregular morphology. Characterization by XRD, DSC, and FTIR spectroscopy collectively demonstrated that QT-NCs represent a thermodynamically stable, novel crystalline form, rather than a physical mixture of QT and GL, consistent with the characteristics of a co-crystal. Compared to the liposomal formulations developed by Lenin Da and Meenaz M. Sayyed et al. [48,49], the QT-NC system exhibits a simpler preparation process, superior environmental friendliness, and an enhanced safety profile. During topical administration, the submicron particle size (200–300 nm) facilitates effective diffusion through the lipid layers of the stratum corneum, while the irregular morphology promotes deeper penetration and prolonged retention within hair follicles [13]. These characteristics collectively make QT-NCs particularly advantageous for treating AGA, a dermatological condition of follicular origin. Ex vivo studies demonstrated that this system significantly enhances skin permeation and retention, achieving 2.27-fold greater cumulative penetration and 2.47-fold higher skin retention compared to raw QT. Importantly, the therapeutic efficacy of the QT-NC gel was validated in a dihydrotestosterone (DHT)-induced AGA mouse model. Topical application significantly improved follicular activity and perifollicular vascularization, as evidenced by earlier pigmentation onset, increased hair coverage, and thicker hair shafts. Molecular analysis revealed downregulation of DHT and 5AR, reduced expression of the negative growth modulator TGF-β1, and upregulation of the angiogenesis- and proliferation-associated markers VEGF, CD31, and Ki67.

The incorporation of GL as a biofunctional stabilizer was critical not only in enhancing nanocrystal stability but also in exerting complementary therapeutic effects [50]. GL has been shown to upregulate IL-10 and α-MSH [15], both involved in creating a favorable follicular microenvironment. Studies have demonstrated that QT can downregulate multiple inflammatory mediators, including TNF-α, IL-1β, and IL-6 [51]. When combined with other active compounds, QT exhibits significant synergistic effects, leading to markedly improved hair coverage and restored skin thickness compared to monotherapy groups. This enhanced efficacy may be attributed to the dual mechanisms of both compounds: while each contributes to anti-inflammatory actions, QT additionally reduces androgen hormone levels, thereby diminishing DHT-induced follicular aggression. Consequently, this combination therapy not only ameliorates the follicular microenvironment to promote regeneration but also reduces the number of atrophic hair follicles.

The current study lacks a comprehensive investigation into the follicular targeting mechanism and therapeutic actions of QT-NCs against androgenetic alopecia. Future work will focus on elucidating the precise mechanisms underlying their follicular targeting efficiency and cutaneous interactions, alongside a more thorough exploration of their pharmacological pathways. Although the efficacy of QT-NCs has been validated in murine models, further blinded studies are required to comprehensively evaluate their safety and effectiveness in larger animal cohorts and human subjects.

## 5. Conclusions

This study successfully developed a QT-NC gel formulation suitable for topical application and evaluated its therapeutic potential for AGA. QT was co-processed with GL using a top–down wet milling strategy to produce nanocrystals, which significantly enhanced dermal permeability and skin retention compared to native QT. Topical administration of the QT-NC gel elicited pronounced anti-androgenic and pro-regenerative effects in a DHT-induced AGA mouse model. Notably, QT and GL exhibited synergistic bioactivity, effectively promoting hair regrowth. These findings suggest that the QT-NC gel could serve as a promising therapeutic option for AGA and offer valuable insights into the rational design of anti-alopecia drug formulations. Future studies should prioritize further investigation into the follicular targeting efficiency and cutaneous interaction mechanisms of QT-NCs. Concurrently, while deepening the understanding of their pharmacological actions, early-phase clinical trials with limited sample sizes should be initiated to preliminarily evaluate translational potential.

## Figures and Tables

**Table 1:** The systematic optimization conditions.

Constraints	Variables	Parameters				
600 rpm, 1.0 h	Mass ratio of QT to GL	8:1	4:1	2:1	1:1	1:2
1∶1, 1.0 h	Milling speed	200 rpm	400 rpm	600 rpm	800 rpm	1000 rpm
1∶1, 200 rpm	Milling time	1.0 h	2.0 h	3.0 h	4.0 h	5.0 h

**Table 2:** Rheological parameter values of the power-law model.

Samples	Consistency Index (K)	Flow Behavior Index (*n*)	R^2^
Carbomer gel	166.594 ± 2.222	0.197 ± 0.004	0.989
QT-NC gel	103.000 ± 1.962	0.202 ± 0.006	0.979

Values are expressed as the mean ± SD (*n* = 3).

**Table 3:** Permeability-related parameters.

Formulation	Q_48_ (μg/cm^2^)	K_p_ (cm/h)	J_ss_ (μg·cm^−2^·h^−1^)	EI
QT gel	28.48 ± 5.55	1.38 ± 0.34	6.89 ± 1.70	2.14
QT-NC gel	64.79 ± 13.45	2.96 ± 0.77 *	14.80 ± 3.83 *	

Values are expressed as the mean ± SD (*n* = 3). *n* = 3; * *p* < 0.05.

## Data Availability

The data presented in this study are available in this article.

## Supplementary Materials

The following supporting information can be downloaded at: https://www.mdpi.com/article/10.3390/pharmaceutics17091188/s1, Figure S1: Specificity Investigation; Figure S2: Standard curve equation for QT. Table S1: Precision Data; Table S2: Repeatability Data.
