# Supplementary material for: Quercetin Nanocrystal Gel: A Novel Topical Therapeutic Strategy for Androgenetic Alopecia
Source: Pharmaceutics. 2025 Sep 12;17(9):1188. doi: 10.3390/pharmaceutics17091188 (PMC12473781; doi:10.3390/pharmaceutics17091188)
Supplement: Supplementary file 1 [file pharmaceutics-17-01188-s001.zip › pharmaceutics-3830244-supplementary.pdf]

Supplementary Materials

Yaya Su, Yuwen Zhu, Lei Ren, Xiang Deng, Rui Song, Lingling Wu, Zhihui Yang * and Hailong Yuan *

Department of Pharmacy, Air Force Medical Center, PLA, Air Force Medical University, Beijing 100142, China
* Correspondence: yangzhihui084045@126.com (Z.Y.); yhlpharm@126.com (H.Y.)

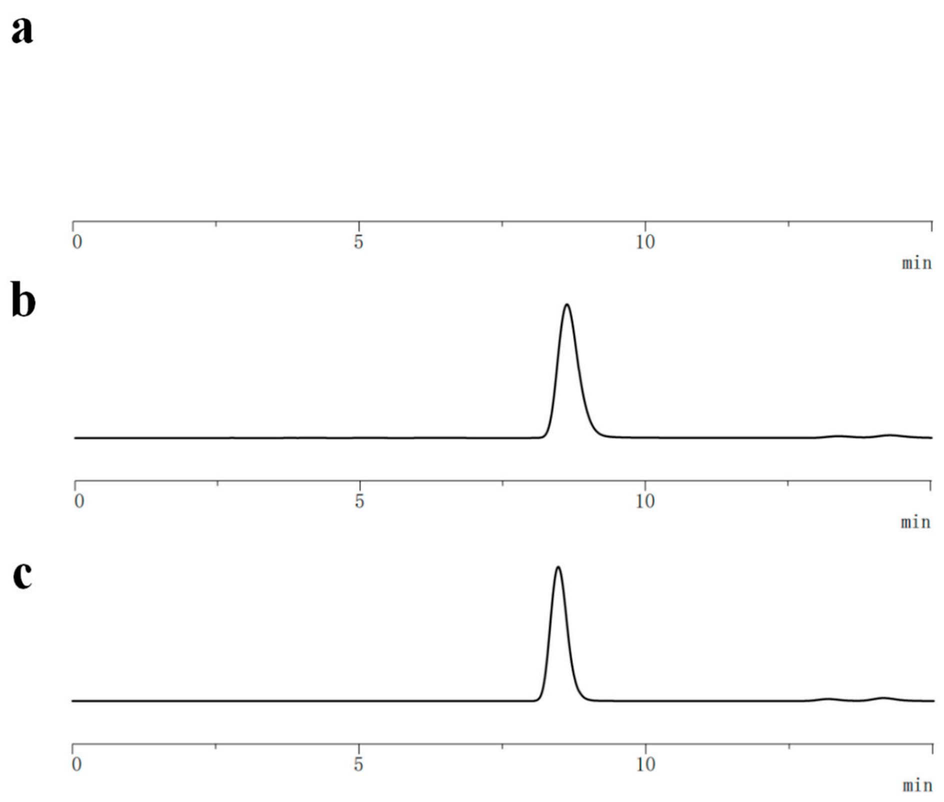

Figure S1. Specificity Investigation: (a) Negative control solution. (b) QT reference standard solution. (c) QT-NCs solution.

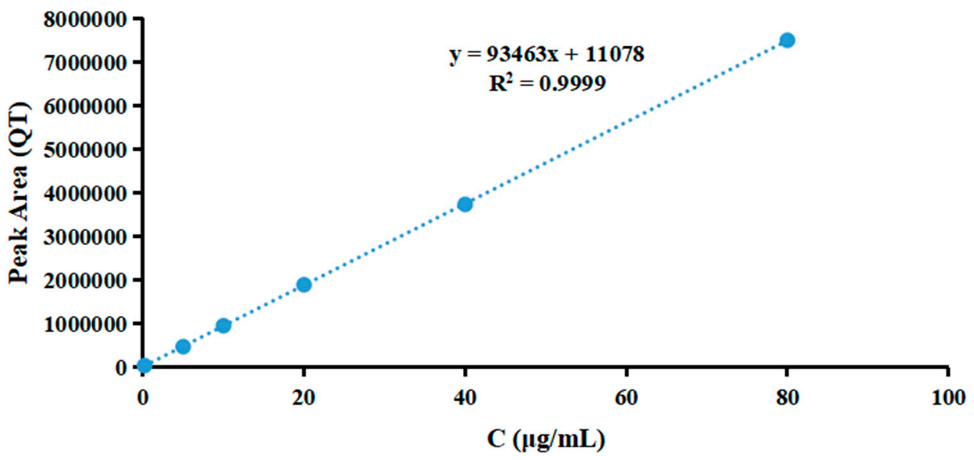

Figure S2. Standard curve equation for QT.

Table S1. Precision Data.

C (μg/mL)	Peak Area (QT)	Mean Peak Area (QT)	RSD
40	2901845		
40	2908517		
40	2905310	2907543	0.18%
40	2908130		
40	2917145		
40	2904311		

Table S2. Repeatability Data.

Sample	Peak Area (QT)	Mean Peak Area (QT)	RSD
1	3017276		
2	3013506		
3	3011055	3011099	0.16%
4	3005295		
5	3005780		
6	3013679		
